# Effect of metabolic genetic variants on long-term disease comorbidity in patients with type 2 diabetes

**DOI:** 10.1038/s41598-021-82276-3

**Published:** 2021-02-02

**Authors:** Shifteh Abedian, Ali Abbasi, Anthonius de Boer, Bruno H. Stricker, Stephan J. L. Bakker, Pim van der Harst, Sanaz Sedaghat, Maryam Darvishian, M. Arfan Ikram, Gerjan Navis, Abbas Dehghan, Ido Pen, Ronald P. Stolk, Harold Snieder, Olaf H. Klungel, Patrick Souverein, Behrooz Z. Alizadeh

**Affiliations:** 1 Department of Epidemiology, University Medical Center Groningen, University of Groningen, Groningen, the Netherlands; 2 Department of Internal Medicine, University Medical Center Groningen, University of Groningen, Groningen, the Netherlands; 3 Division of Pharmacoepidemiology and Clinical Pharmacology, Utrecht Institute for Pharmaceutical Sciences (UIPS), Utrecht University, Utrecht, the Netherlands; 4 Department of Epidemiology, Erasmus Medical Center Rotterdam, Rotterdam, the Netherlands; 5 Department of Cardiology and Thorax Surgery and Experimental Cardiology, University Medical Center Groningen, Groningen, the Netherlands; 6 School of Population and Public Health, University of British Columbia, Vancouver, BC Canada; 7 Theoretical Research in Evolutionary Life Sciences, Groningen Institute for Evolutionary Life Sciences, University of Groningen, Groningen, the Netherlands; 8 Julius Center for Health Sciences and Primary Care, University Medical Center Utrecht, Utrecht, the Netherlands

**Keywords:** Genetics, Diseases, Medical research

## Abstract

Underlying genetic determinants contribute to developing type 2 diabetes (T2D) future diseases. The present study aimed to identify which genetic variants are associated with the incident of the major T2D co-morbid disease. First, we conducted a discovery study by investigating the genetic associations of comorbid diseases within the framework of the Utrecht Cardiovascular Pharmacogenetic studies by turning information of > 25 years follow-up data of 1237 subjects whom were genotyped and included in the discovery study. We performed Cox proportional-hazards regression to examine associations between genetic variants and comorbid diseases including cardiovascular diseases (CVD), chronic eye disease, cancer, neurologic diseases and chronic kidney disease. Secondly, we replicated our findings in two independent cohorts consisting of 1041 subjects. Finally, we performed a meta-analysis by combining the discovery and two replication cohorts. We ascertained 390 (39.7%) incident cases of CVD, 182 (16.2%) of chronic eye disease, 155 (13.8%) of cancer, 31 (2.7%) of neurologic disease and 13 (1.1%) of chronic kidney disease during a median follow-up of 10.2 years. In the discovery study, we identified a total of 39 Single Nucleotide Polymorphisms (SNPs) associated with comorbid diseases. The replication study, confirmed that rs1870849 and rs8051326 may play a role in the incidence of chronic eye disease in T2D patients. Half of patients developed at least one comorbid disease, with CVD occurring most often and earliest followed by chronic eye disease. Further research is needed to confirm the associations of two associated SNPs with chronic eye disease in T2D.

## Background

Type 2 diabetes (T2D) is an increasing global health issue. There are currently about 420 million people live with T2D worldwide^1,2^. Up to 80 percent of patients with diabetes experience higher rates of morbidity and premature mortality due to macrovascular and microvascular complications of diabetes, such as cardiovascular disease (CVD), nephropathy, retinopathy and neuropathy^3^. Evidence suggests that early glycaemic control in patients with T2D is associated with a reduction in complications of diabetes^3^. Glycemic control may be achieved by adherence to a healthy lifestyle and using hypoglycemic agents. Inter-individual variation in developing complications of diabetes is attributed to differences in patients’ demographics, lifestyle factors, medication adherence, and genetic factors^4^.

T2D and its related comorbid diseases are caused by the interplay of both genetic and environmental factors over the lifespan^5^. Data from genome-wide association studies (GWAS) have to date identified more than 200 Single Nucleotide Polymorphisms (SNPs) that are associated with T2D and metabolic traits^6^. Similarly, GWAS has identified many genetic variants that are implicated with CVD^7,8^. Clinical and observational evidence support the importance of T2D as a major risk factor for CVD along with dyslipidemia and hypertension^9^. T2D increases the risk of CVD by two- to fourfold^10^ and CVD is the cause of death for about half of patients with T2D^11^. There is genetic susceptibility to comorbid diseases associated with T2D, such as CVD and cancer^12^.

Yet, the role of individual genetic variations as determinants of major micro- and macrovascular complications remains to be understood. In this study, we hypothesized that certain genotypes may prone patient with T2D to development of co-morbid conditions. We aimed to examine the associations between genetic variants and the incidence of major co-morbid diseases related to diabetes, including CVD, cancer, chronic kidney disease, chronic eye disease and neurological diseases. In the present naturalistic study, we report the incidence of disease comorbidities with T2D in a cohort of 1237 patients who were followed up over 10 years by the means of hospital records and registries from pharmacies. We next conducted a discovery study to investigate the genetic signal for comorbid diseases in these patients with T2D using a gene-centric ITMAT-Broad-CARe” (IBC) 50 k SNP Illumina array chip that includes ~ 50,000 genetic variants from ~ 2100 loci. Finally, we sought to replicate our novel associated variants in two independent cohorts.

## Methods

### Study settings

The study was carried out in two stages. First, we conducted a discovery study by investigating the genetic associations of comorbid diseases. Secondly, we replicated our findings in two independent cohort studies. Finally, we performed a meta-analysis using a random-effects model by combining information from the discovery and two replication cohorts.

### Study population

We performed a historical-cohort design supplemented with a longitudinal data analysis of the disease records of patients with T2D. The diagnosis of diabetes was defined via: (1) use of at least one anti-diabetic drug (either oral hypoglycemic agents or insulin in combination with oral hypoglycemic agents or as the second line for treatment) based on pharmacy records; (2) self-reported having T2D in questionnaire. Cases identified by self-report method were verified against pharmacy records. For this discovery study, data from the Hospitalisation Database linked to the Out-patient Pharmacy Database were used. Controls were matched to the cases on age (± 2 years), sex, and region. They were assigned the same index date as the cases to whom they were matched. The discovery stage was conducted within the framework of the Utrecht Cardiovascular Pharmacogenetic studies (UCP) (Supplementary Text S1). UCP comprises subjects derived from the PHARMO Database Network (a population-based network of electronic healthcare databases). The PHARMO Database Network combines data from different primary and secondary healthcare settings in the Netherlands (Supplementary Text S2).

From this source population, a total sample of 1237 prevalent or incident cases of T2D was recruited and genotyped. Data over the outcomes were collected during the study period between 1985 and 2010 including prevalent and incident of T2D. We defined prevalent cases of diabetes as those who had reported or registered as having T2D either before 1985 (start of PHARMO registry) or before being included in the UCP study (n = 377). Patients who indicated they had diabetes before the study period in the questionnaires associated with the UCP project were considered as prevalent cases. Any subject who reported or registered as having T2D during or after being included in the UCP was defined as an incident case of T2D (n = 860). We replicated our findings in two independent cohorts consisting of Rotterdam Study (Supplementary Text S3) and Prevend Study (Supplementary Text S4).

All participants gave written informed consent prior to study inclusion. The study protocol conforms to the ethical guidelines of the 1975 Declaration of Helsinki as reflected in approval by the medical ethical review board of the University Medical Center Groningen, the Netherlands.

### Co-morbid diseases

We extracted all records of diseases that occurred in both prevalent and incident T2D patients. We defined five diabetes-related complications or co-morbid diseases including the occurrence of CVD, chronic eye disease, cancer, neurologic disease and chronic kidney disease, which happened after diagnosis of T2D from 1985 to 2010. We used the International Classification of Diseases (ICD-9) to define and classify diagnosis of each comorbid disease. Supplementary Table S1 shows the list of ICD-9 diagnoses for definition of each incident outcome during follow-up.

We ascertained incidence of a specified comorbid disease (e.g. a patient with CVD), if a T2D patient developed the comorbid disease (e.g. incidence of CVD) after diagnosis of T2D. Thus, out of 1237 included subjects, persons who did not have a history of comorbid disease (e.g. remained free of CVD), were considered as non-cases (i.e. reference set) for the specified outcome (i.e. CVD). The same procedure was performed to ascertain other outcomes including chronic eye disease, cancer, neurologic disease, and chronic kidney disease. In the case of CVD, given the nature of UCP for cardiovascular diseases, a number of patients had myocardial infarction (MI) and were included as cases in the original case–control studies of HYPERGEN and STATGEN and some patients developed CVD multiple times during study period. We thus defined recurrent comorbidity of CVD when a T2D patient who had a medical history of having one or more occurrences of CVD before the diagnosis of T2D, developed CVD again after diagnosis of T2D.

Given we applied a historical-cohort design, we included both prevalent and incident cases of T2D, and also the registered comorbid diseases between 1985 and 2010. We defined four scenarios for each comorbid disease separately (Supplementary Figure S2). The first scenario (S1) included T2D patients who never experienced the studied comorbid disease across their lifespan by the end of follow-up (i.e., 2010). This group was used as the persons free from the outcome for association analyses for the given comorbid disease. The second scenario (S2) included T2D patients who had registered as having comorbid diseases both before and after diagnosis of T2D (i.e. they had multiple recurrent comorbid diseases). This scenario was most seen in T2D patients who also had CVD diseases. The third scenario (S3) included T2D patients who had registered as having a comorbid disease only after the diagnosis of T2D and before the end of follow-up. The fourth scenario (S4) included T2D patients who had received a diagnosis of a comorbid disease only before the diagnosis of T2D (Supplementary Figure S2).

In longitudinal analyses, comorbidity of a disease with T2D was considered if a T2D patient developed a comorbid disease before and after (i.e. recurrent, S2), or only after (S3) the diagnosis of T2D.

### Genotyping

Participants were genotyped by using the custom ITMAT-Broad-CARe (IBC) 50 k SNP Illumina array. Genotype calling was done using a Genome Studio calling algorithm. This is a cardiovascular gene-centric array designed for large-scale genomic association studies. In brief, this array contains high-density SNPs (> 51,000) across more than 2000 candidate genes within pathways believed to underpin primary and secondary cardiovascular and metabolic disease processes. Details of this array have been described previously^16^ and in Supplementary Text S1. Supplementary Figure S1 depicts the flow of the quality control process. These SNPs are included in the analysis as potential candidate variants underlying the comorbidity of diseases in T2D.

### Data analysis

Data were shown as mean ± standard deviation (SD) or median interquartile range (IQR) for continuous variables and frequency (%) for categorical variables.

To study the association of each SNP with each of the comorbid diseases, we pooled T2D patients with S1, S2, and S3 together and excluded patients of S4 (i.e. those who had comorbid disease only before the diagnosis of T2D). We modeled Cox regression comparing cases defined as individuals from scenarios S2 or S3 as T2D patients with first incidence or recurrence of comorbid disease with individuals from S1 who were T2D patients with no recorded comorbid disease (i.e. reference set) (Supplementary Figure S2). To define the underlying time-scale, we used the time of diagnosis of T2D as the start of follow-up (t0) and time-to-event was calculated as the length of the time interval between t0 and the time of recurrence (S2) or first incidence of comorbid disease (S3) occurred. We used Cox proportional-hazards regression models to examine the association of qualified SNPs with the incidence of each comorbid disease as specified above. In adjusted models, hazard ratios (HRs) and 95% confidence interval (CI) for each outcome were calculated for SNPs that passed quality control. We fitted models adjusted for age, sex, birth cohort (i.e. each decade from date of birth coded as 1 for 1900–1910; 2 for 1911–1920, and so on, and five ancestry eigenvectors from principal components (PCs) analysis which was necessary to control for population stratification. All analyses were performed using additive genetic models and results were reported per each copy of the minor allele. By correcting for multiple testing, a *P* value equal to or less than 10^–4^ was considered statistically significant. All statistical analyses were conducted using SPSS IBM 19, PLINK, and R version 2.15.0.

Given the number of the tests that were performed and given potential enrichment of signal from outcomes based on a priori selection of SNPs on this chip, quantile-.quantile (Q-Q) plots were used to show possible inflation of statistics. This analysis indicates whether observed associations potentially deviate from the expected distribution under the null hypothesis. Q-Q plots present the observed 2log_e_ *P* value calculated for all SNPs plotted against the expected 2log_e_ *P* value for χ2 statistics. When there was no inflation noted, we calculated the genomic control coefficient, called lambda (λ), to quantify the potential effects of population structure on the observed association. A λ value close to 1.0 indicates minimal inflation of statistics due to uncommon variants, heterogeneity, or sub-population.

### Replication study

We selected the significantly associated variants for any of the five outcomes in the discovery study. Then we tested replication of these variants in two other independent prospective longitudinal cohorts, including the Rotterdam Study and Prevend cohort (Supplementary Text 2 describes the cohorts), that had available longitudinal data of studied comorbid diseases in T2D patients, and co-variables, except for neurological diseases. We applied Cox proportional hazards regression analysis as described for the discovery stage to the data from replication cohorts. We extracted summary statistics as HR and SE for the studied SNPs. In total, we included information of 733 subjects with CVD, 505 with chronic eye disease, 422 with cancer, and 87 patients with chronic kidney disease. Meta-analysis was performed by pooling of HR using random effects by combining the discovery and two replication cohorts. Random effects models were conservatively used to corroborate any potentially undetected source of heterogeneity in our analysis, given we might have not been able to detect true subpopulation differences between study individuals. We tested for heterogeneity using I^2^. We defined statistical significance as *P* < 0005 of results for replication.

## Results

### Baseline characteristics of participants

Table 1 shows the baseline characteristics of the participants. The mean duration of T2D was 10.4 (SD 8.5) years. The median follow-up time to first incidence of comorbid disease ranged from 8.5 (CVD) to 9.9 (chronic kidney disease) years after diagnosis of T2D.

**Table 1:** Baseline characteristics of incident and prevalent patients with T2D with genetic information available in UCP.

	Incident cases of T2D (n = 860)	Prevalent cases of T2D (n = 377)
Age-mean ± SD	51.58 ± 20.66	57.18 ± 19.96
Male-n. (%)	564 (65.6)	253 (67.1)
Age_sex check-no. (%) Correct	842 (98.2)	367 (97.3)
Met QC n. (%)	774 (90.0)	335 (88.9)
Questionnaire n. (%) Data available	822 (95.6)	367 (97.3)
**Questionnaire information at T2D diagnosis**		
*BMI (Kg/m^2^) ± SD	27.7 ± 11.0	
**Smoking-n. (%)**		
Current	273/822 (31.7)	
Past	289/822 (33.6)	
Alcohol consumption -n. (%)	453/827 (52.7)	
Physical activity (leisure time) < 4-h /week- n. (%)	423/837 (49.1)	
Hypertension-n. (%)	527/854 (61.3)	
Hyperlipidemia-n. (%)	322/854 (37.4)	

Data were shown as mean ± Standard deviation (SD).Categorical variables are shown as frequencies (percentage).

*BMI: body mass index (the weight in kilogram divided by the square of the height in metres).

### Comorbidity study

In the total sample of 1237 patients with T2D who had data, we observed that 415 (33.5%) T2D patients had at least one co-occurrence of CVD, 215 (17.4%) had a chronic eye disease, 168 (13.6%) had cancer. There were 34 patients (2.7%) with neurologic disease and 14 (1.1%) with chronic kidney disease (Table 2).

**Table 2:** Incidences of comorbid diseases in patient with T2D in UCP.

	Total T2D patients (n = 1237)	Incident cases of T2D (n = 860)	Prevalent cases of T2D (n = 377)
**Incidences of comorbid diseases after T2D diagnosis**			
*Cardiovascular disease	415 (33.54)	231 (26.86)	184 (48.80)
IHD-no.(%)	303 (24.5)	158 (18.4)	145 (38.5)
Hypertension-no. (%)	12 (1.0)	8 (0.9)	4 (1.1)
Cerebrovascular-no. (%)	64 (5.2)	40 (4.6)	24 (6.4)
CABG/PCI-no. (%)	107 (8.6)	47 (5.5)	60 (15.9)
Peripheral artery disease-no. (%)	61 (4.9)	34 (4.0)	27 (7.2)
Peripheral artery intervention-no. (%)	68 (5.5)	40 (4.7)	28 (7.4)
Chronic eye disease-no. (%)	215 (17.4)	109 (12.7)	106 (28.1)
Cancer-no.(%)	168 (13.6)	106 (12.3)	62 (16.4)
Neurologic disease	34 (2.7)	19 (2.2)	15 (4.0)
Chronic kidney disease -no. (%)	14 (1.1)	6 (0.7)	8 (2.1)
**First occurrence of comorbid diseases (new or recurrent event before or after diagnosis of T2D)**			
*Cardiovascular disease	572 (46.24)	385 (44.76)	187 (49.60)
IHD-no.(%)	476 (38.4)	327 (38.0)	149 (39.5)
Hypertension-no. (%)	21 (1.0)	17 (2.0)	4 (1.1)
Cerebrovascular-no. (%)	96 (7.7)	72 (8.4)	24 (6.4)
CABG/PCI-no. (%)	166 (13.4)	105 (12.2)	61 (16.2)
Peripheral artery disease-no. (%)	92 (7.4)	64 (7.4)	28 (7.4)
Peripheral artery intervention-no. (%)	97 (7.8)	69 (8.0)	28 (7.4)
Chronic eye disease-no. (%)	215 (17.4)	109 (12.7)	106 (28.1)
Cancer-no.(%)	216 (17.4)	153 (17.8)	63 (16.7)
Neurologic disease-no. (%)	47 (3.7)	32 (3.7)	15 (4.0)
Chronic kidney disease-no. (%)	15 (1.0)	7 (0.8)	8 (2.1)

We ascertained incident cases of a specified comorbid disease if a patient developed the comorbid disease after a diagnosis of T2D.

*: Cardiovascular disease was diagnosed if a patient had at least one of these comorbid diseases. The number of CVD is less than the summary of this comorbid disease because some of the patients had more than one comorbid disease.

### Genetic study

In the discovery study, we applied Cox proportional-hazards models to 38,997 genotyped SNPs in 1163 patients with T2D. Results are presented in Supplementary Tables S2. There was no evidence of spurious inflation of *P* value as the Q-Q plots showed the expected distribution (Supplementary Figures S3). The observed genetic associations for each outcome are presented by Manhattan plots (Supplementary Figures S3) showing *P* values for each tested variant. Supplementary Table S2 presents the strongest associations observed between 39 variants and each outcome.

### Cardiovascular disease

In the discovery analysis out of 1237 patients with T2D, 1163 patients passed quality control, of whom 182 subjects (with self-reported CVD) had either no verified data (by checking hospital admission reports) on CVD or time of CVD incidence or had CVD only before the diagnosis of T2D (S4: 19 cases), were excluded from the analysis. The remaining 962 subjects were included in the analysis as 25 patients having a recurrence of CVD before and after T2D diagnosis (S2) or 415 patients with CVD after diagnosis of T2D (S3), who were compared to 522 T2D patients without any experience of CVD (S1). During a median (IQR) follow-up of 7 (3 to 12) years, we ascertained 390 incident cases of CVD; yielding an incidence rate of 39.7%. Five SNPs reached the significance threshold of less than *P* < 1 × 10^–4^ using Cox regression models for incident CVD. The most strongly associated SNP was the rs3796164*G allele with an effect allele frequency (EAF) of 0.04 in T2D patients with CVD and 0.01 in the reference set yielding a HRs (s.e; *P* value) of 2.92 (0.24; 7.95 × 10^−6^) per each effect allele (Supplementary Table S2a). In the replication analysis, only three SNPs out of five SNPs were available in two replication cohorts. These SNPs were not significantly associated with CVD incidence in the replication cohorts (Table 3a). The meta-analysis also failed to find any significant association of these SNPs with CVD incidence, when the data were combined (Table 3a).

**Table 3:** Meta-analysis of cohorts for the association of suggestive SNPs with the comorbid disease in T2D patients.

a CVD				Discovery 390/981		Replication				Meta-analysis 772/2012		
						Prevend 39/143		Rotterdam 343/888				
SNP	Chr: Position	Risk Allele	MAF	HR (95%CI)	*P* val	HR (95%CI)	*P* Val	HR (95%CI)	*P* Val	P HR (95%CI)	*P* Val	*P* Val het
rs1288331	1:53,403,637	G	0.42	1.34 (1.17–1.54)	2.49 × 10^–5^	1.43 (0.91–2.24)	0.13	1.01 (0.86–1.17)	0.90	1.21 (0.95–1.53)	0.11	0.01
rs7553128	1:53,384,490	A	0.21	1.45 (1.21–1.72)	5.17 × 10^–5^	2.14 (0.70–6.54)	0.18	0.87 (0.72–1.05)	0.16	1.22 (0.77–1.92)	0.39	0.00
rs13415601	2:210,788,369	A	0.07	0.54 (0.41–0.72)	2.76 × 10^–5^	na	–	0.88 (0.67–1.15)	0.35	0.69 (0.43–1.10)	0.12	0.01

b Chronic eye disease				Discovery 182/1124		Replication				Meta-analysis 505/1974		
						Prevend		Rotterdam 323/850				
SNP	Chr: Positon	Risk Allele	MAF	HR (95%CI)	*P* val	HR (95%CI)	*P* Val	HR (95%CI)	*P* Val	P HR (95% CI)	*P* Val	*P* Val het
rs10464834	8:97,640,442	A	0.16	2.19 (1.51–3.19)	8.55 × 10^–5^	na	–	1.05 (0.80–1.37)	0.69	1.50 (0.73–3.09)	0.26	0.00
rs11036364	11:5,205,580	A	0.42	0.63 (0.50–0.78)	4.02 × 10^–5^	na	–	1.08 (0.92–1.26)	0.30	0.83 (0.48–1.41)	0.49	0.00
rs1870849	16:81,380,132	A	0.08	1.87 (1.36–2.55)	1.02 × 10^–5^	na	–	1.40 (0.97–2.00)	0.06	1.64 (1.23–2.17)	0.00	0.23
rs8051326	16:81,380,213	G	0.08	1.89 (1.35–2.63)	1.15 × 10^–5^	na	–	1.40 (0.97–2.00)	0.06	1.64 (1.22–2.19)	0.00	0.23

c Cancer				Discovery 155/1119		Replication				Meta-analysis 444/2157		
						Prevend 22/141		Rotterdam 267/897				
SNP	Chr: Position	Risk Allele	MAF	HR (95%CI)	*P* val	HR (95%CI)	*P* Val	HR (95%CI)	*P* Val	P HR (95%CI)	*P* Val	*P* Val het
rs4576663	1:190,123,195	A	0.09	2.43 (1.60–3.66)	2.66 × 10^–5^	1.64 (0.05–45.92)	0.77	0.82 (0.52–1.30)	0.41	1.43 (0.54–3.77)	0.45	0.00
rs2305948	4:55,979,558	A	0.13	1.90 (1.39–2.61)	3.97 × 10^–5^	0.29 (2.71–33.11)	0.62	0.99 (0.72–1.37)	0.97	1.34 (0.73–2.47	0.34	0.01
rs1882149	12:119,922,525	A	0.15	1.87 (1.39–2.50)	5.68 × 10^–5^	0.69 (0.01–39.68)	0.86	0.85 (0.64–1.13)	0.27	1.24 (0.60–2.56)	0.56	0.00

d Chronic kidney disease				Discovery 13/1159		Replication				Meta-analysis 87/1950		
						Prevend		Rotterdam 74/791				
SNP	Chr: Position	Risk Allele	MAF	HR (95%CI)	*P* val	HR (95%CI)	*P* Val	HR (95%CI)	*P* Val	P HR (95% CI)	*P* Val	*P* Val het
rs742829	6:151,300,463	G	0.46	7.76 (2.86–21.11)	6.39 × 10^–5^	na	–	1.30 (0.85–2.00)	0.21	3.00 (0.52–17.1)	0.21	0.00
rs249	8:19,855,286	G	0.30	5.16 (2.26–11.75)	8.09 × 10^–5^	na	–	0.79 (0.38–1.63)	0.53	2.00 (0.32–12.51)	0.45	0.00
rs640090	10:95,382,575	G	0.15	9.49 (3.23–27.91)	4.65 × 10^–5^	na	–	2.31 (0.29–17.93)	0.42	6.20 (1.74–22.08)	0.00	0.23
rs4281621	14:61,025,684	G	0.15	11.79 (4.34–32.07)	1.63 × 10^–6^	na	–	2.29 (0.71–7.38)	0.16	5.35 (1.07–26.65)	0.04	0.03
rs1110192	14:61,061,727	A	0.19	15.64 (5.01–48.76)	1.89 × 10^–6^	na	–	2.41 (0.75–7.72)	0.13	6.17 (0.99–38.47)	0.05	0.02
rs7159886	14:61,030,897	G	0.15	11.04 (3.34–36.48)	9.35 × 10^–5^	na	–	2.14 (0.66–6.88)	0.19	4.84 (0.97–24.09)	0.05	0.05

### Chronic eye disease

From 1163 T2D patients that passed quality control, 39 subjects that had either no verified data on chronic eye disease or time of incidence or reported that only before the diagnosis of T2D, were excluded from this analysis. The remaining 1124 subjects were included in the analysis as 0 patients having chronic eye disease before and after T2D diagnosis (S2) or 215 patients with chronic eye disease only after the diagnosis of T2D (S3), who were compared to 909 T2D patients without any history of chronic eye disease (S1). During a median (IQR) follow-up of 7 (4 to 12) years, we ascertained 182 incident cases of chronic eye disease; yielding an incidence of 16.2%. Among the nine SNPs which reached the significant threshold of *P* < 1 × 10^–4^, the rs1870849*A allele showed the most significant association with incident chronic eye disease (EAF of 0.08 in cases and 0.05 in the reference set yielding a HR of 1.89 (0.17) per effect allele (*P* = 1.02 × 10^−5^); (Supplementary Table S2b). Combined with the replication study, the meta-analysis showed a significant association to chronic eye disease with a combined HR of 1.64 [95%CI 1.23–2.17] for rs1870849 and 1.64 [95%CI 1.22–1.96] for rs8051326 (Table 3b).

### Cancer

From 1163 T2D patients that passed quality control, 44 subjects that had either no verified data on cancer or time of cancer incidence or had cancer only before the diagnosis of T2D, could not be analysed. The remaining 1119 subjects were included in the analysis as 48 patients having cancer before and after T2D diagnosis (S2) or 168 patients with cancer only after diagnosis of T2D (S3), who were compared to 903 T2D patients without any history of cancer (S1). During a median follow-up of 7 (3 to 12) years, we ascertained 155 incident cases of cancer; yielding an incidence of 13.8%. Nine SNPs reached the significant threshold of less than *P* < 1 × 10^–4^ using Cox regression models (Table 3c). The most strongly associated SNPs were rs4576663 and rs1882149 for risk of incident cancer. The EAF of rs4576663*A was 0.08 in T2D patient with cancer compared to that of 0.04 in the reference set. In the multivariable-adjusted model, SNPs rs3796164 and rs1882149 remained significantly associated with risk of incident cancer events, with HRs (s.e; *P* value) of 2.43 (0.21; 2.66 × 10^−5^) and 1.87 (0.15; 5.68 × 10^−5^), respectively (Supplementary Table S2c). In the replication analysis, only rs1882149, and two other SNPs (rs2305948 and rs4576663) had available data across the three cohorts. These SNPS were not significantly associated with incidence of cancer in T2D patients (Table 3c). Likewise, the meta-analysis did not show any significant association for any of these SNPs when the data were combined (Table 3c).

### Neurologic disease

From 1163 T2D patients that passed quality control, 4 subjects that had either no verified data on neurologic disease or time of incidence or reported that only before the diagnosis of T2D, could not be analysed. The remaining 1159 subjects were included in the analysis as 13 patients having neurologic disease before and after T2D diagnosis (S2) or 34 patients with a neurologic disease only after the diagnosis of T2D (S3), who were compared to 1112 T2D patients without any history of neurologic disease (S1). During a median follow-up of 8 (4 to 12) years, we ascertained 31 incident cases of neurologic disease; yielding an incidence of 2.7%. In the discovery study, association analyses found six SNPs with a *P* < 1 × 10^–4^ being rs11600557*A the most significant variant with EAF of 0.12 in patients versus EAF of 0.27 in the reference set leading to an HR (s.e.; *P* value) of 6.87 (0.42; 4.83 × 10^−6^) per effect allele (Supplementary Table 2d). There was no data available to perform a replication analysis for this outcome in the replication cohorts.

### Chronic kidney disease

From 1163 T2D patients that passed quality control, four subjects that had either no verified data on chronic kidney disease or time of incidence or had chronic kidney disease only before the diagnosis of T2D, could not be analysed. The remaining 1159 subjects were included in the analysis as 1 patient having chronic kidney disease before and after T2D diagnosis (S2) or 14 patients with only after the diagnosis of T2D (S3), who were compared to 1144 T2D patients without any history of chronic kidney disease (S1). During a median follow-up of 8 (5 to 13) years, we ascertained 13 incident cases of chronic kidney disease; yielding an incidence of 1.1%. Ten SNPs reached the significant threshold of *P* < 1 × 10^–4^. The most strongly associated SNPs were rs4281621*G (EAF in the cases 0.19 versus (vs.) 0.02 in the reference set) and rs1110192*A (EAF in the cases 0.19 vs. 0.02 in the reference set) for risk of incident kidney disease in T2D patients. In the multivariable-adjusted model, SNPs rs4281621 with inflated HRs (s.e; *P* value) of 11.80 (0.51; 1.63 × 10^−6^) and rs1110192 with HR 15.64 (0.58; 1.89 × 10^−6^) were significantly associated with the risk of incident kidney disease, (Supplementary Table S2e). These two SNPs along with the other five significantly associated SNPs were available in two replication cohorts and were included in the replication study. None of these SNPs were replicated at nominal significance (Table 3d). The meta-analysis of combined data across the three cohorts, also failed to verify the initial observed significant associations from discovery analysis.

## Discussion

In this study, we showed that CVD is the most common comorbid condition associated with T2D, followed by chronic eye disease and cancer. Next, we aimed to identify potential genetic causes of these comorbidities in T2D. In a two-stage design, we investigated associations of almost 40,000 candidate variants associated with cardio-metabolic diseases residing on a 50 k gene-centric array. At the discovery stage, we found 39 SNPs to be associated with the incidence of cardiovascular disease, chronic eye disease, cancer, neurologic disease, or chronic kidney disease in T2D patients, employing Cox-proportional hazard analysis. In the replication study, none of these variants were replicated, except two variants which remained associated with the incidence of chronic eye disease in T2D.

### The Co-morbidity of complications in T2D

T2D often occurs together with other complex diseases, which complicates the management of the disease, adversely affects quality of life and leads to early mortality. One cohort study that followed patients during 1999–2004 showed that only 14% of T2D patients had no comorbidities^13,14^. Our study found CVD had the highest cumulative incidence of 33% and was the first complication to occur after diagnosis of T2D. Most previous studies that have estimated the incidence of T2D comorbidities, also reported CVD as having the highest cumulative incidence after diabetes diagnosis^15^.

T2D is the most common leading cause of comorbid chronic and end-stage kidney disease with changes in prevalence and clinical characteristics worldwide and Western countries. More than 50% of T2D patients worldwide develop chronic kidney disease with a higher risk of complications, decreasing quality of life, and premature mortality^16^_._

In epidemiological evidence, T2D is increasingly recognized as a risk factor for colorectal, kidney, pancreatic, liver, gallbladder, breast, ovary, cervix, several digestive diseases, endometrial cancer, and non-Hodgkin's lymphoma^17,18^. A combination of several shared risk factors such as diet, high BMI, less physical activity may explain this association between diabetes and cancer. There is evidence that hyperinsulinemia, hyperglycemia, and inflammation following diabetes might be responsible for the link between diabetes and cancer risk in T2D patients^19^. The original role of different anti-diabetic medications in increasing the risk of cancer is still unclear. One study showed that among antidiabetic users, metformin decreased the risk of prostate cancer compared to other antidiabetic medications^20^_._ Another study demonstrated that monotherapy with metformin was significantly associated with reduced risk of developing pancreatic cancer and hepatocellular carcinoma compared with taking other anti-diabetic medication such as insulin and sulphonylurea, but not for colon and breast cancer^21^. In another study, compared with non-insulin antidiabetic medication, insulin increased the risk of colorectal and pancreatic cancer^22^_._

### The genetic study

We found two SNPs including rs1870849 (synonym: rs58631131) and rs8051326 associated with the incidence of chronic eye diseases in T2D patients. According to homo sapiens annotation release 108, these variants are common transcript variants occurring in an intron region of an uncharacterized long non-coding RNA (LOC101928446) mapped on chromosome 18, within the vicinity of the Cadherin 13 (CDH13; known as CDHH; P105) gene. CDH13 is translated to 14 transcripts due to alternative splicing. The gene encodes a member protein of the cadherin superfamily. The protein is anchored by a GPI moiety to the surface of the cell membrane. This protein presents two functions of acting as a negative regulator of axon growth during neural differentiation and protecting vascular endothelial cells from apoptosis due to oxidative stress. T-cadherin is highly expressed in the vascular system especially in small blood vessels, and neurons of the brain cortex and spinal cord. Though the pathologies related to cadherin function remain unclear, some data suggest its role in atherosclerotic lesions and conditions associated with pathological angiogenesis. Given the potential role of small vessel plasticity in the pathogenesis of retinal disease in T2D patients, our findings support the hypothesis that CDH13, and in general the CDH superfamily may play a role in eye pathologies in T2D. Our association findings remain to be confirmed in future studies, and testing the hypothesis of the relation of CDH13 with eye diseases requires more functional studies.

In our discovery analysis, we found rs3796164 as the most strongly associated variant for CVD and cancer, which is annotated close to the MYLK gene. Although this SNP was not replicated, the Human MYLK gene is an attractive candidate for cardiovascular diseases. It encodes isoforms of myosin-activating protein kinase (MLCK), which is responsible for muscle contraction, including smooth, cardiac, and skeletal muscle cells^23,24^. Cardiac MLCK plays a critical role in the cardiogenesis and normal cardiac function. Microarray analysis of failing human cardiac cells and animal models has implicated MLCK expression in the pathogenesis of heart failure via its potential biological role in contractility of myocardium^23–25^. For MYLK, common variants in intronic regions have been associated with inflammatory lung diseases, as well as the risk of asthma and coronary artery disease^26–28^.

Likewise, the other two common variants associated with cancer in the discovery analysis, namely rs1882149 and rs7961178, are located in the intergenic region of the Hepatocyte Nuclear Factor-1alpha (HNF1A) gene. At HNF1A, some common and rare signals have been associated with low-density lipoprotein (LDL) cholesterol, circulating C-reactive protein levels, and risk of monogenic diabetes (maturity-onset diabetes of the young type 3) or multi-factorial T2D^29–31^. This gene encodes four hepatocyte nuclear factors (HNF) which play important roles in the development and regeneration of the liver^32^. HNF1α is a transcription factor that is involved in the regulation and expression of many other genes such as those in the liver^32,33^. The allelic or somatic mutations in HNF1A for hepatic cancer such as adenoma and hepatocellular carcinoma (HCC; inactivated gene in 35% of HCC cases)^33,34^ are well-known. In addition, the risk of other types of cancer, like pancreatic cancer or prostate cancer can be affected by genetic alterations in the family of HNF^35^. However, these SNPs were not replicated. In fact, non-replication is an issue in observational studies mostly attended to the power of the included replication cohorts, small sample size, or a different sample selection processes, underlying heterogeneity of the replication cohort, presence of confounding, inappropriate analytical approach, and false positivity from the initial discovery study. In our study, we had a small sample size of outcomes as our replicating cohort were taken from the general population, and thus participant of these cohorts are at a lower risk of complications associated with T2D, compared to our discovery study, hence yield an attenuated genetic effect on the disease in healthy people. Though we did not replicate these SNPs, this gene is an interesting candidate for the risk of incident cancer in T2D patients. This might explain the potential biological link between diabetes and cancer.

### Study limitations and strengths

We obtained data on pharmacy and hospital admission records linked to questionnaires which allowed us to identify patients with T2D and ascertain clinical outcomes. We defined incident cases via ICD9 coding for all T2D patients, to control phenotypic heterogeneity. Next, we were able to estimate the effects of genetic variants on the risk of outcomes when the time of onset of disease was known. In other words, we reported HRs for outcomes per effect allele, while assessment of the genetic associations is usually expressed in an odds ratio, as is usual in case–control design studies^1,3,4,36^.

We had selected T2D samples that had risk factors for developing hard outcomes out of three initial studies. This selection of T2D patients may enhance the association of risk variants with the incidence of hard outcomes, and hence increase the power of our discovery study where we observed several significant associations of SNP with hard outcomes. When we performed replication analysis of the T2D patients selected out of population-based cohort studies, we failed to replicate our findings. Combining high-risk samples selected for T2D trials with those selected out of T2D cohort studies may impose limitations to our study, attenuating the chance of replication of our findings. Nevertheless, given we performed a case-only design, covering patients with T2D, the effect estimates of comorbidity study and genetic study seem not to have affected the results much, given the prevalence of hard outcomes were comparable across the three groups of patients across HYPERGEN, UDES, and STATGEN.

There are also some limitations to our study. We showed that CVD is the most common comorbid condition associated with T2D. When we compare to data from other cohorts, our report might be overestimated, given the UCP (including HYPERGEN and STATGEN), including individuals at a higher risk for CVD. We recruited data from T2D patients who were nearly all adults (while the number of children with T2D was very low), and our findings should be replicated in other populations. For example, we used the PREVEND and Rotterdam Studies as a replication study, which contains few subjects with T2D and the duration of T2D was very short to develop the desired outcomes of the study. This is more appealing when we consider diabetes care in the Netherlands is among the best in the world, with consequences for the risk of developing complications. Our estimates from replication cohorts might be underestimated, and impose a limitation for the generalizability of the results to other countries. Although our genotype data had certain limitations regarding the availability of the number of SNPs, we used a platform incorporating candidate genes to examine whether those variants were potentially associated with the risk of multifactorial diseases^5,8,13^.

Because we included data only from chronic outcomes which were recorded in the database and ascertained by ICD codes, we might have missed other comorbid disease in T2D patients. Moreover, we would expect that the size of our cohort of T2D patients in combination with the low incidence of some of the outcomes (i.e. low study power) like kidney disease led to a slight underestimation of the observed risk per effect allele. The other issue may be originated from naturalistic design as the outcomes (T2D and CVD) are indications for the admissions in hospital, leading to a selection bias towards patients for high risk in CVD, or our study was embedded within cohorts of patients selected for mainly CVD, thus more risk for severe events. This could lead to an underestimation of the effect size and thus negative results especially for less frequent outcomes such as kidney disease.

### Conclusion

We showed that in the course of the last half-century, comorbid diseases including CVD, chronic eye disease, and cancer, remain the most common complications of T2D. There is not much evidence that the incidences of cardiometabolic diseases are strongly determined by genetic variants, but our data suggest that rs1870849 and rs8051326 and their annotated gene CDH13 may play a role in the incidence of chronic eye disease in T2D patients. Overall, these results do not provide strong evidence that common variants underlie the incidence of comorbid conditions in T2D. However, associations for these SNPs variants warrant further investigation with larger studies.

## Supplementary Information

Supplementary Information.

## Abbreviations

T2D: Type 2 diabetes
UCP: Utrecht Cardiovascular Pharmacogenetic study
CVD: Cardiovascular disease
SNPs: Single Nucleotide Polymorphisms
GWAS: Genome-wide association studies
IBC: ITMAT-Broad-CARe
ACS: Acute coronary syndrome
AMI: Acute myocardial infarction
ISD: International Classification of Diseases
IHD: Ischemic heart disease
MI: Myocardial infarction
S1: The first scenario
S2: Second scenario
S3: Third scenario
S4: Fourth scenario
SD: Standard deviation
IQR: Interquartile range
HRs: Hazard ratios
CI: Confidence interval
PCs: Principal components
Q-Q: Quantile-.quantile
λ: Lambda
EAF: Effect allele frequency
vs.: Versus
MLCK: Myosin-activating protein kinase
HNF1A: Hepatocyte Nuclear Factor-1alpha
LDL: Low-density lipoprotein cholesterol
HNF: Hepatocyte nuclear factors
HCC: Hepatocellular carcinoma

## References

[1] Qi L (2011). Genetic susceptibility to coronary heart disease in type 2 diabetes: 3 independent studies. J. Am. Coll. Cardiol..

[2] Williams WW (2012). Association testing of previously reported variants in a large case-control meta-analysis of diabetic nephropathy. Diabetes.

[3] Fowler MJ (2011). Microvascular and macrovascular complications of diabetes. Clin. Diab..

[4] Qi Q (2012). Genetic variants, plasma lipoprotein (a) levels, and risk of cardiovascular morbidity and mortality among two prospective cohorts of type 2 diabetes. Eur. Heart J..

[5] Sobrin L (2011). Candidate gene association study for diabetic retinopathy in persons with type 2 diabetes: the Candidate gene Association Resource (CARe). Investig. Ophthalmol. Vis. Sci..

[6] Frau F (2017). Type-2 diabetes-associated variants with cross-trait relevance: Post-GWAs strategies for biological function interpretation. Mol. Genet. Metab..

[7] Varshney A (2017). Genetic regulatory signatures underlying islet gene expression and type 2 diabetes. Proc. Natl. Acad. Sci..

[8] Deloukas P (2013). Large-scale association analysis identifies new risk loci for coronary artery disease. Nat. Genet..

[9] Emerging Risk Factors Collaboration (2011). Diabetes mellitus, fasting glucose, and risk of cause-specific death. N. Engl. J. Med..

[10] Go AS (2014). Executive summary: heart disease and stroke statistics—2014 update: a report from the American Heart Association. Circulation.

[11] Di Angelantonio E (2015). Association of cardiometabolic multimorbidity with mortality. JAMA.

[12] Manolio TA, Brooks LD, Collins FS (2008). A HapMap harvest of insights into the genetics of common disease. J. Clin. Investig..

[13] Keating BJ (2008). Concept, design and implementation of a cardiovascular gene-centric 50 k SNP array for large-scale genomic association studies. PLoS ONE.

[14] Suh D-C (2010). Impact of comorbid conditions and race/ethnicity on glycemic control among the US population with type 2 diabetes, 1988–1994 to 1999–2004. J. Diab. Its Compl..

[15] Seguchi O (2007). A cardiac myosin light chain kinase regulates sarcomere assembly in the vertebrate heart. J. Clin. Investig..

[16] Tsilidis KK (2015). Type 2 diabetes and cancer: umbrella review of meta-analyses of observational studies. BMJ.

[17] Castillo JJ (2012). Increased incidence of non-Hodgkin lymphoma, leukemia, and myeloma in patients with diabetes mellitus type 2: a meta-analysis of observational studies. Blood J. Am. Soc. Hematol..

[18] Starup-Linde J (2013). CARING (CAncer Risk and INsulin analoGues): the association of diabetes mellitus and cancer risk with focus on possible determinants-a systematic review and a meta-analysis. Curr. Drug Saf..

[19] Haring A (2017). Antidiabetic drug use and prostate cancer risk in the Finnish Randomized Study of Screening for Prostate Cancer. Scand. J. Urol..

[20] DeCensi A (2010). Metformin and cancer risk in diabetic patients: a systematic review and meta-analysis. Cancer Prev. Res..

[21] Karlstad O (2013). Use of insulin and insulin analogs and risk of cancer—systematic review and meta-analysis of observational studies. Curr. Drug Saf..

[22] Horne BD (2009). Validation study of genetic associations with coronary artery disease on chromosome 3q13-21 and potential effect modification by smoking. Ann. Hum. Genet..

[23] Lazar V, Garcia JGN (1999). A single human myosin light chain kinase gene (MLCK; MYLK) transcribes multiple nonmuscle isoforms. Genomics.

[24] Asakura M, Kitakaze M (2009). Global gene expression profiling in the failing myocardium. Circ. J..

[25] Han YJ (2012). An intronic MYLK variant associated with inflammatory lung disease regulates promoter activity of the smooth muscle myosin light chain kinase isoform. J. Mol. Med..

[26] Gao L (2007). Polymorphisms in the myosin light chain kinase gene that confer risk of severe sepsis are associated with a lower risk of asthma. J. Allergy Clin. Immunol..

[27] Thomas MC, Cooper ME, Zimmet P (2016). Changing epidemiology of type 2 diabetes mellitus and associated chronic kidney disease. Nat. Rev. Nephrol..

[28] Voight BF (2010). Twelve type 2 diabetes susceptibility loci identified through large-scale association analysis. Nat. Genet..

[29] Kathiresan S (2009). Common variants at 30 loci contribute to polygenic dyslipidemia. Nat. Genet..

[30] Ridker PM (2008). Loci related to metabolic-syndrome pathways including LEPR, HNF1A, IL6R, and GCKR associate with plasma C-reactive protein: the Women's Genome Health Study. Am. J. Hum. Genet..

[31] Zeng X (2011). Recombinant adenovirus carrying the hepatocyte nuclear factor-1alpha gene inhibits hepatocellular carcinoma xenograft growth in mice. Hepatology.

[32] Jeannot E (2012). Double heterozygous germline HNF1A mutations in a patient with liver adenomatosis. Diab. Care.

[33] Zucman-Rossi J (2006). Genotype–phenotype correlation in hepatocellular adenoma: new classification and relationship with HCC. Hepatology.

[34] Li D (2012). Pathway analysis of genome-wide association study data highlights pancreatic development genes as susceptibility factors for pancreatic cancer. Carcinogenesis.

[35] Sandholm N (2012). New susceptibility loci associated with kidney disease in type 1 diabetes. PLoS Genet..

[36] Abbasi A (2012). Prediction models for risk of developing type 2 diabetes: systematic literature search and independent external validation study. BMJ.

