# Supplementary material for: Effect of metabolic genetic variants on long-term disease comorbidity in patients with type 2 diabetes
Source: Sci Rep. 2021 Feb 2;11:2794. doi: 10.1038/s41598-021-82276-3 (PMC7854581; doi:10.1038/s41598-021-82276-3)
Supplement: Supplementary file 1 — Supplementary Information. [file 41598_2021_82276_MOESM1_ESM.pdf]

Effect of metabolic genetic variants on long-term disease comorbidity in patients with type 2 diabetes

Shifteh Abedian[1]; Ali Abbasi[1,2]; Anthonius de Boer[3]; Bruno H. Stricker[4]; Stephan JL Bakker[2]; Pim van der Harst[5]; Sanaz Sedaghat[4]; Maryam Darvishian[6]; M. Arfan Ikram[4]; Gerjan Navis[2]; Abbas Dehghan[4]; Ido Pen[7]; Ronald P. Stolk[1]; Harold Snieder[1]; Olaf H Klungel[3,8]; Patrick Souverein[3]; Behrooz Z. Alizadeh[1]

[1]Department of Epidemiology, University of Groningen, University Medical Center Groningen, Groningen, the Netherlands.
[2]Department of Internal Medicine, University of Groningen, University Medical Center Groningen, Groningen, the Netherlands.
[3]Division of Pharmacoepidemiology & Clinical Pharmacology, Utrecht Institute for Pharmaceutical Sciences (UIPS), Utrecht University, the Netherlands.
[4]Department of Epidemiology, Erasmus Medical Center Rotterdam, Rotterdam, the Netherlands.
[5]Department of Cardiology and Thorax Surgery & Experimental Cardiology, University Medical Center Groningen, Groningen, the Netherlands.
[6]School of Population and Public Health, University of British Columbia, Vancouver, BC, Canada.
[7]Theoretical Research in Evolutionary Life Sciences, Groningen Institute for Evolutionary Life Sciences, University of Groningen, Groningen, the Netherlands.
[8]Julius Center for Health Sciences and Primary Care, University Medical Center Utrecht, the Netherlands.

Correspondence to:

Shifteh Abedian MD, PhD (s.abedian@umcg.nl)
Department of Epidemiology
University Medical Center Groningen
Groningen, The Netherlands

Supplementary Text and Figures

Overview of contents

1- Supplementary Text S1: UCP Study, p3
2- Supplementary Text S2: PHARMO Database Network, p3
3- Supplementary Text S3: Rotterdam Study, p3
4- Supplementary Text S4: Prevend Study, p4
5- Supplementary Text S5: Quality check of genetic data, p4
6- Supplementary Tables S, p5- 8
   1. Supplementary Table S1: ICD-9 codes for definition of comorbid disease p5
   2. Supplementary Tables S2: Association of variants with the risk of comorbid disease in patients with T2D in discovery cohort (p6-8)
7- Supplementary Figures S, p9-15
   1. Supplementary Figure S1: Overview of the quality control of genotype data (p9)
   2. Supplementary Figure S2: Scenarios of T2D related comorbid disease (p9)
   3. Supplementary Figures S3: QQ plots and Manhattan plots of the genome-wide significant associated SNPs for comorbid disease (p11-15)
8- References, p16

Supplementary Text S1

The UCP Study The Utrecht Cardiovascular Pharmacogenetic study (UCP) comprises subjects derived from the PHARMO Database Network. In short, patients with a high cardiovascular risk defined as those with a dispensing for an antihypertensive drug[37], or a glucose lowering drug[38], or who had hypercholesterolemia (prescription for a cholesterol-lowering drug or total cholesterol 0>5.0 mmol/l[39]). From this cohort, patients hospitalized for acute coronary syndrome (ACS) were included as cases acute myocardial infarction (AMI), International Classification of Diseases 9 (ICD-9) code 410 or sub-acute forms of IHD (ICD-9 codes 411.1 and 411.8) were included as cases if they were registered in the PHARMO Database Network for at least one year.

UCP included three components UDES (patients who started using diabetic medication between 1991 until 2004; n=298), HYPERGEN (case-control study among antihypertensive drug users; n=953) and STATGEN (Hypercholesterolemia patients using statins; n=149).

Supplementary Text S2

The PHARMO Database Network The PHARMO Database Network combines data from different primary and secondary healthcare settings in the Netherlands (www.pharmo.nl). At the time of study, the base population of PHARMO Database covered approximately 2,000,000 community-dwelling inhabitants of several population-defined areas in the Netherlands.

Participants were recruited through community pharmacies, where they received a letter in which the purpose of the study was explained. They were asked to return an informed consent form and a filled-out questionnaire. After the participant had consented to participate in the study, (s) he was sent an Oragene collection kit (hypercholesterolemic and diabetic cohort), or three cotton swabs and tubes containing buffer (hypertensive cohort) to collect saliva. All participants were explicitly asked to consent for the collection, storage and genotyping of the DNA material. Approval for this study was obtained from the Medical Ethics Committee of the University Medical Center Utrecht, The Netherlands.

Supplementary Text S3

The Rotterdam Study The Rotterdam Study is a prospective population-based cohort study of chronic diseases in the old people which started in 1990. The study population consists of 14,926 cases aged 45 years or over living in the city of Rotterdam in The Netherlands. The main objectives of the Rotterdam Study were to examine the risk factors of cardiovascular, neurological, ophthalmological and endocrine diseases that are frequent in in the old people. The study was designed as a response to the demographic changes that were important to an increase of the proportion of old people in most populations. The findings of the Rotterdam Study have been presented in over 1500 research articles and reports (see www.erasmus-epidemiology.nl/rotterdamstudy)[40].

Supplementary Text S4:

The Prevend Study Prevend study (Prevention of Renal and Vascular End- stage Disease), running in the city of Groningen, the Netherlands. This study is an ongoing Dutch prospective population-based cohort study for which 8592 participants (age range, 28-75 years) that were recruited between 1997 and 1998[41, 42]. This study follows relation of increasing level of urinary albumin excretion and its relation to renal and cardiovascular disease[43].

Supplementary Text S5:

The quality check of genetic data We applied a parallel standard quality control method to filter out low quality samples and SNPs as depicted in the flow diagram in supplementary figure 1. Regarding participants, we first excluded 34 subjects lacking data on age and sex as well as 43 with genotyping failure on more than 50% of SNPs. Next, we excluded 24 subjects due to genotyping failure of more than 10% of SNPs, and 154 with sex mismatch between registry records and genetic data of the X chromosome as well as three subjects who were found to be duplicate records or related individuals (i.e., sibs) through identity-by-descent (IBD) analysis of complete genetic data with a PI-HAT value of greater than or equal to 0.5. The remaining 1,185 participants with T2D were used to examine the association of genetic variants in a full cohort design. Regarding SNPs, 1,163 variants were excluded due to a missing rate of more than 50% of study subjects, and of the remaining variants, another 786 variants with a missing rate of more than 10%. After exclusion of rare SNPs with a minor allele frequency of less than 1%, 38,997 SNPs passed quality control for analysis. The sample of 1163 T2D patients were selected and included to genotype –phenotype association analysis using hazard model.

Supplementary Table S1: ICD-9 codes for definition of comorbid disease

CARDIOVASCULAR DISEASE	
Ischemisch heart disease	410-414
cerebrovascular disease	430–438
diseases of arteries, arterioles, capillaries	440–445
peripheral angiopathy	443.81
operations on vessels of heart	36
**CANCER**	140-239
**EYE DISEASES**	
blindness	369.00-369.9
cataract	366.41
glaucoma	365.44
macular edema	362.07
retinal edema	362.07
retinopathy	362.01-362.07
**CHRONIC KIDNEY DISEASE**	585.1-585.9
nephropathy NOS	583.81
nephrosis	581.81
intercapillaryglomerulosclerosis	581.81
kimmelstiel-Wilson syndrome	581.81
**NEUROLOGIC DISEASE**	
mononeuropathy	354.0-355.9
neurogenic arthropathy	713.5
peripheral autonomic neuropathy	337.1
polyneuropathy	357.2

Supplementary Table S2. Association of variants with the risks of comorbid diseases in patients with T2D in the discovery cohort

a CVD

SNP	Risk allele	Chr: Position	MAF	HR (S.E)	P value	Nearest Gene/locus	Type of variants
rs1288331	G	1:53403637	0.42	1.35 (0.07)	2.49×10^{-5}	SLC1A7	intergenic_variant
rs7553128	A	1:53384490	0.21	1.45 (0.09)	5.17×10^{-5}	SLC1A7	5KB_upstream_variant
rs13415601	A	2:210788369	0.07	0.55 (0.14)	2.76×10^{-5}	ACADL	intron_variant
rs3796164	G	3:124935751	0.04	2.92 (0.24)	7.95×10^{-6}	MYLK	non_synonymous_
rs353648	C	11:35146865	0.11	0.64 (0.12)	10^{-4}	CD44	intron_variant

SNP: Single-Nucleotide Polymorphism**,** MAF: Minor Allele frequency, HR: Hazard Ratio, S.E: Standard Error,
Nearest Gene/locus: the closest Gene or locus to associated SNPs with comorbid disease.
From 981 T2D patients included in scenario 1 to 3, we ascertained 390 (39.7%) incidence cases of cardiovascular disease.

b Chronic eye disease

SNP	Risk allele	Chr: Position	MAF	HR (S.E)	P value	Nearest Gene/ locus	Type
rs237874	G	3:8757338	0.26	2.19 (0.18)	10^{-4}	CAV3	intron_ variant
rs1040655	G	6:71009938	0.12	2.24 (0.18)	10^{-4}	COL9A1	intron_ variant
rs10464834	A	8:97640442	0.16	2.20 (0.19)	8.55×10^{-5}	SDC2	intron_ variant
rs11036364	A	11:5205580	0.42	0.63 (0.11)	4.02×10^{-5}	HBB	intron_ variant
rs8181793	A	13:113545319	0.11	1.79 (0.15)	10^{-4}	GAS6	2KB_upstream_variant
rs1870849	A	16:81380132	0.08	1.87 (0.16)	1.02×10^{-5}	CDH13	intron_ variant
rs8051326	G	16:81380213	0.08	1.89 (0.17)	1.15×10^{-5}	CDH13	intron_ variant
cnvi0085739	N	16:81378779	0.07	0.62 (0.13)	2.10×10^{-5}	N/A	near-gene-5[NM_000820.1]
rs35962914	G	17:10244481	0.02	1.49 (0.11)	2.70×10^{-5}	MYH8 LOC100128560	non_synonymous_codon

*From 1124 T2D patients included in scenario 1 to 3, we ascertained 182 (16.2%) incidence cases of Chronic eye disease.

c Cancer

SNP	Risk allele	Chr: Position	MAF	HR (S.E)	P value	Nearest Gene/ locus	Type
rs4576663	A	1:190123195	0.09	2.43 (0.21)	2.66×10^{-5}	FAM5C	intron[NM_199051.1]
rs2305948	A	4:55979558	0.13	1.91 (0.16)	3.97×10^{-5}	KDR	missense [NM_002253.1]
rs17244376	A	9:139784861	0.04	3.59 (0.33)	10^{-4}	TRAF2	intron[NM_021138.3]
rs7129187	A	11:42227297	0.24	1.67 (0.13)	10^{-4}	LOC387761	intron[XM_373495.4]
rs1882149	A	12:119922525	0.15	1.87 (0.15)	5.68×10^{-5}	HNF1A	intron[NM_000545.4]
SNP12-119924646	A	12:119924646	0.15	1.87(0.15)	5.68×10^{-5}	NA	NA
rs7961178	A	12:119890027	0.15	1.87 (0.16)	9.89×10^{-5}	HNF1A-AS1	5KB_downstream_variant
SNP12-119912205	A	12:119912205	0.16	1.82 (0.15)	10^{-4}	NA	NA
rs17312115	A	23:71686119	0.10	1.83(0.15)	3.99×10^{-5}	HDAC8	intron_ variant

From 1119 T2D patients included in scenario 2 to 3, we ascertained 155 (13.8%) incidence cases of cancer.

d Neurologic disease

SNP	Risk allele	Chr: Position	MAF	HR (S.E.)	P value	Nearest Gene/ locus	Type
rs10494521	A	1:177529955	0.06	8.86 (0.56)	8.67×10^{-5}	SOAT1	intron[NM_003101.4]
rs16873301	A	6:12823579	0.27	2.98 (0.28)	10^{-4}	PHACTR1	5KB_upstream_variant
rs12255719	G	10:83964067	0.21	3.47 (0.31)	6.78×10^{-5}	NRG3	intron[NM_001010848.2]
rs11600557	A	11:511302	0.12	6.87 (0.42)	4.83×10^{-6}	LOC100128703	intron[XM_001714014.1]
rs10841496	C	12:20412921	0.25	0.31 (0.30)	10^{-4}	LOC100131677	intron[XM_001723042.1]
rs11465836	A	X:152935534	0.10	4.04 (0.33)	2.64×10^{-5}	IRAK1	intron_ variant

*From 1159 T2D patients included in scenario 1 to 3, we ascertained 31 (2.7%) incidence cases of Neurologic disease.

e Chronic kidney disease

SNP	Risk allele	Chr: Position	MAF	HR (S.E.)	P value	Nearest Gene/ locus	Type
rs742829	G	6:151300463	0.46	7.77 (0.51)	6.39×10^{-5}	MTHFD1L	intron[NM_015440.3]
rs249	G	8:19855286	0.30	5.16 (0.42)	8.09×10^{-5}	LPL	intron[NM_000237.2]
rs640090	G	10:95382575	0.15	9.50 (0.55)	4.65×10^{-5}	PDE6C	intron[NM_006204.3]
rs4281621	G	14:61025684	0.15	11.80(0.51)	1.63×10^{-6}	PRKCH	intron[NM_006255.3]
rs1110192	A	14:61061727	0.19	15.64 (0.58)	1.89×10^{-6}	PRKCH	intron[NM_006255.3]
rs7159886	G	14:61030897	0.15	11.04 (0.61)	9.35×10^{-5}	PRKCH	intron[NM_006255.3]
rs6609051	G	X:39823169	0.31	3.69 (0.30)	1.42×10^{-5}	BCOR	intron_ variant
rs6629761	A	X:23822068	0.43	3.32 (0.31)	10^{-4}	APOO	intron_ variant_
rs6629758	A	X:23819066	0.43	3.30 (0.31)	10^{-4}	APOO	intron_ variant
rs6629756	A	X:23814013	0.43	3.18 (0.30)	10^{-4}	APOO	intron_ variant

*From 1159 T2D patients included in scenario 1 to 3, we ascertained 13 (1.1%) incidence cases of Chronic kidney disease.

Supplementary Figure S1: Overview of the quality control of genotype data

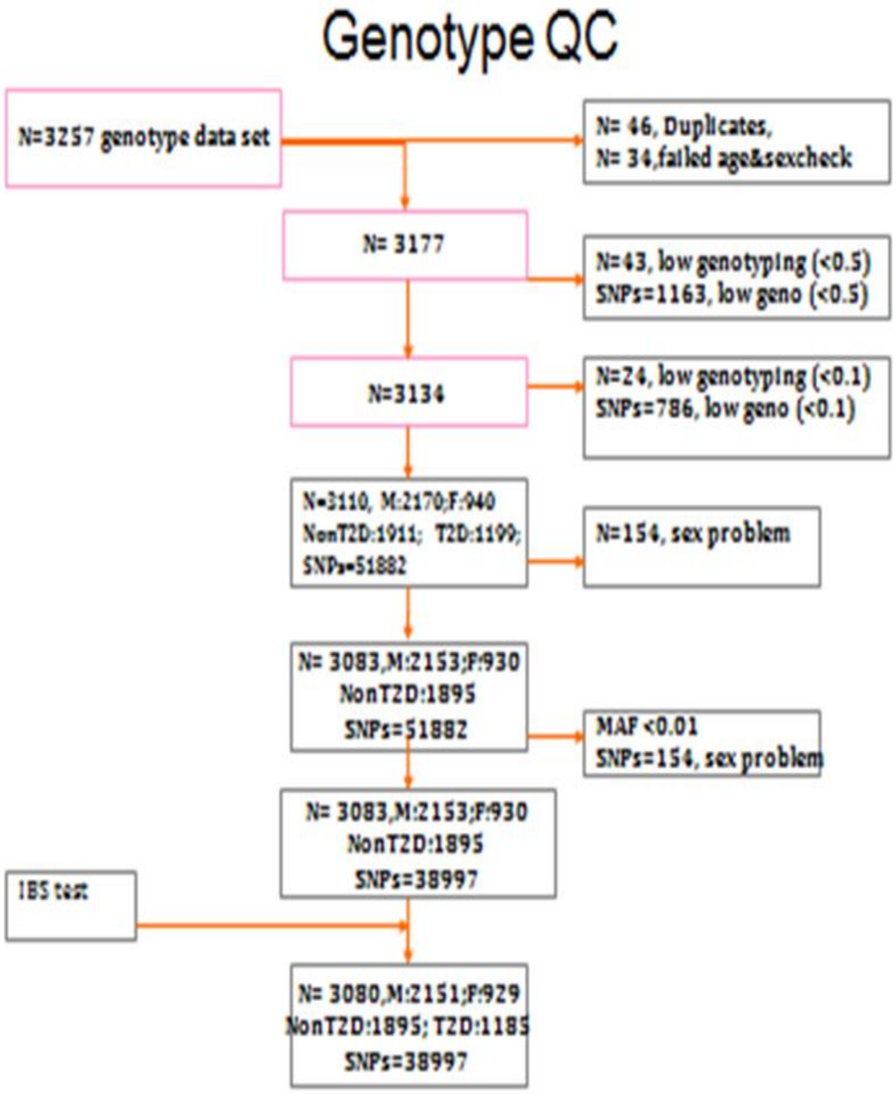

Supplementary Figure S2: Scenarios of T2D-related comorbid disease

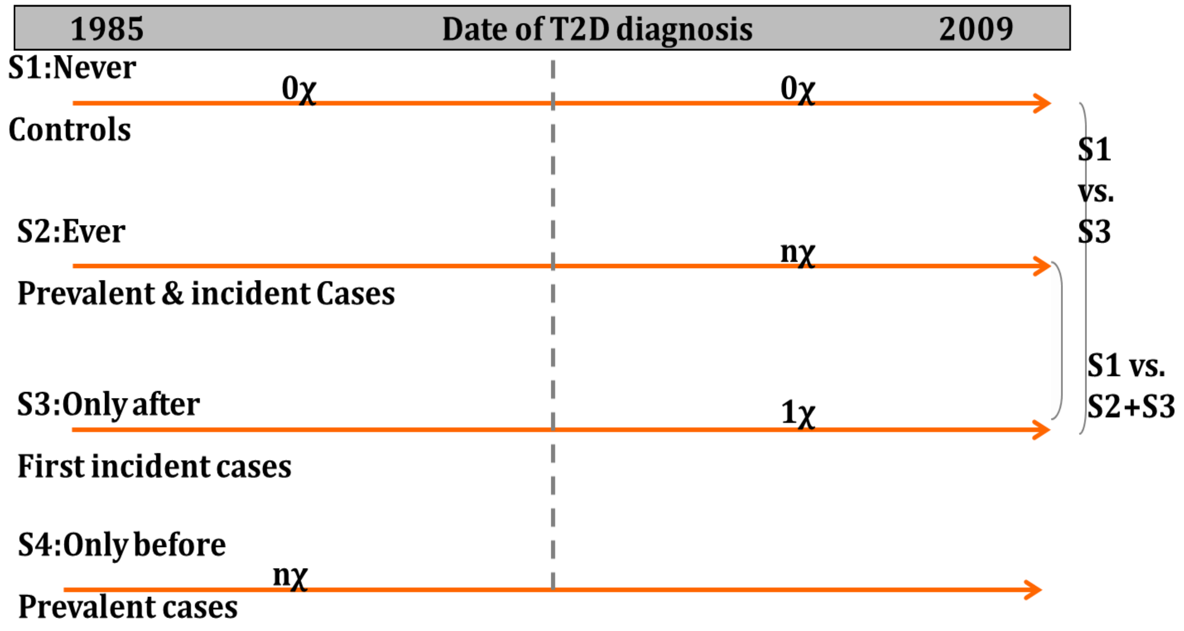

We defined four scenarios for each comorbid disease separately (Supplementary Figure S2). The first scenario (S1) included T2D patients who never experienced the studied comorbid disease across lifespan by the end of follow-up (ie, 2010). This group was set as reference set for association analyses for the given comorbid disease. The second scenario (S2) included T2D patients who had registered as having comorbid diseases both before and after diagnosis of T2D (ie, they had multiple recurrent of comorbid disease. This scenario was most seen in T2D patients with also CVD diseases. Scenario three (S3) included T2D patients who had registered as having a comorbid disease only after the diagnosis of T2D and before the end of follow-up. Scenario four (S4) included T2D patients who had received a diagnosis of a comorbid disease only before the diagnosis of T2D.

Supplementary Figures S3: Manhattan plots and QQ plots of the genome-wide significant associated SNPs for comorbid disease.

a Cardiovascular disease

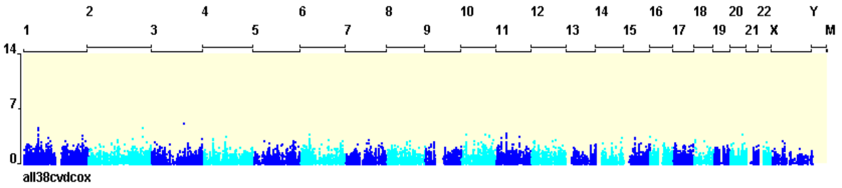

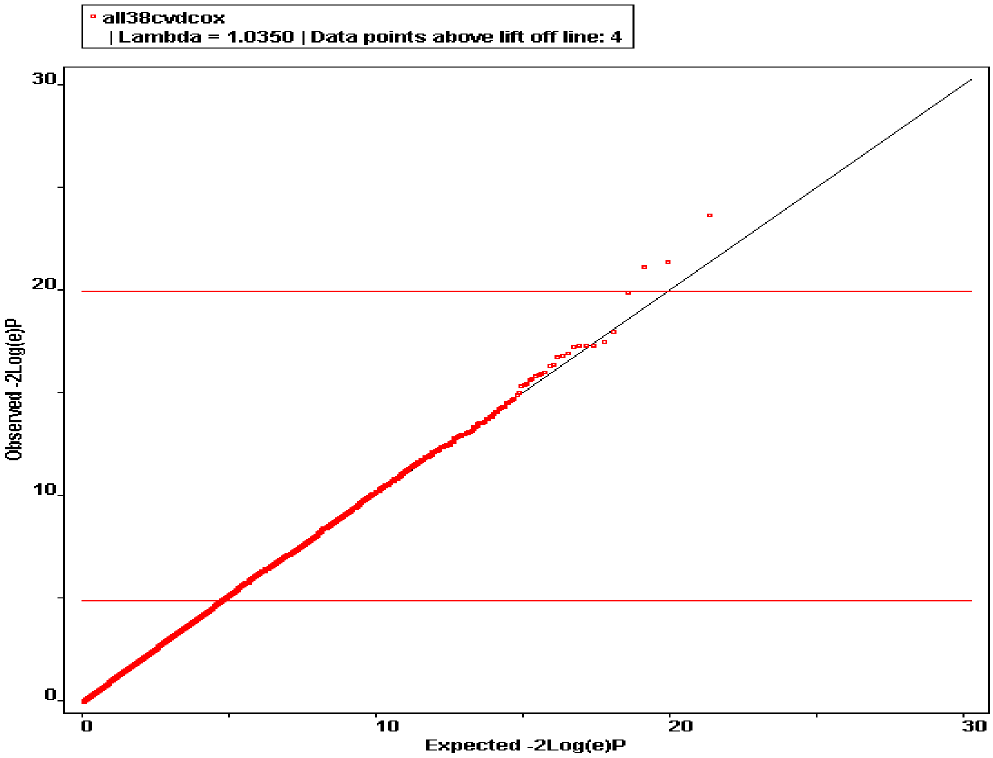

b Chronic Eye disease

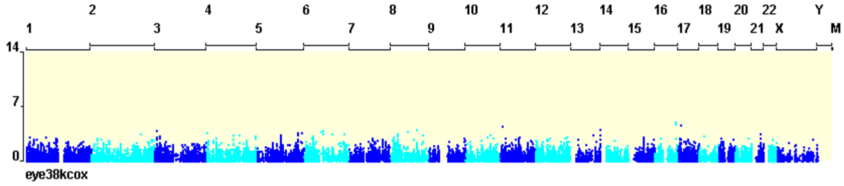

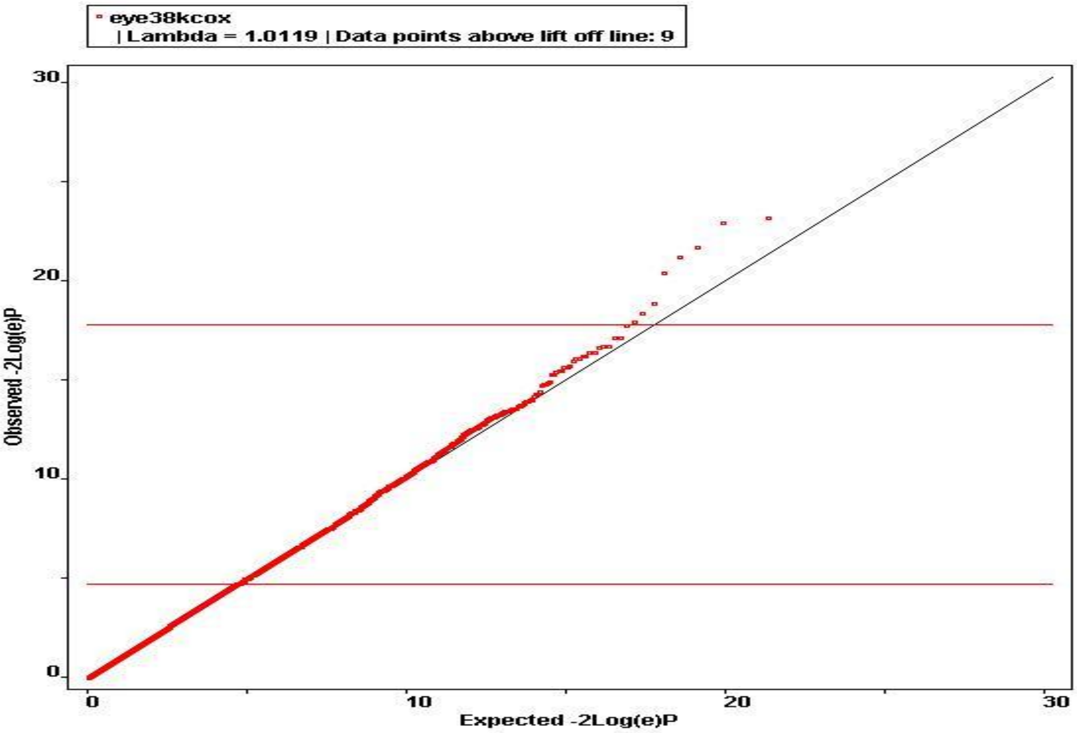

c Cancer

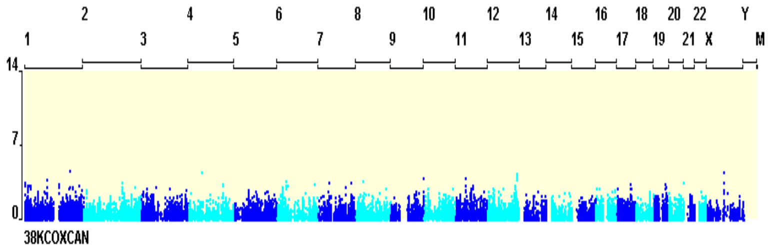
2 4 6 8 10 12 14 16 18 20 22 Y
1 3 5 7 9 11 13 15 17 19 21 X M
14
7
0
38KCOXCAN

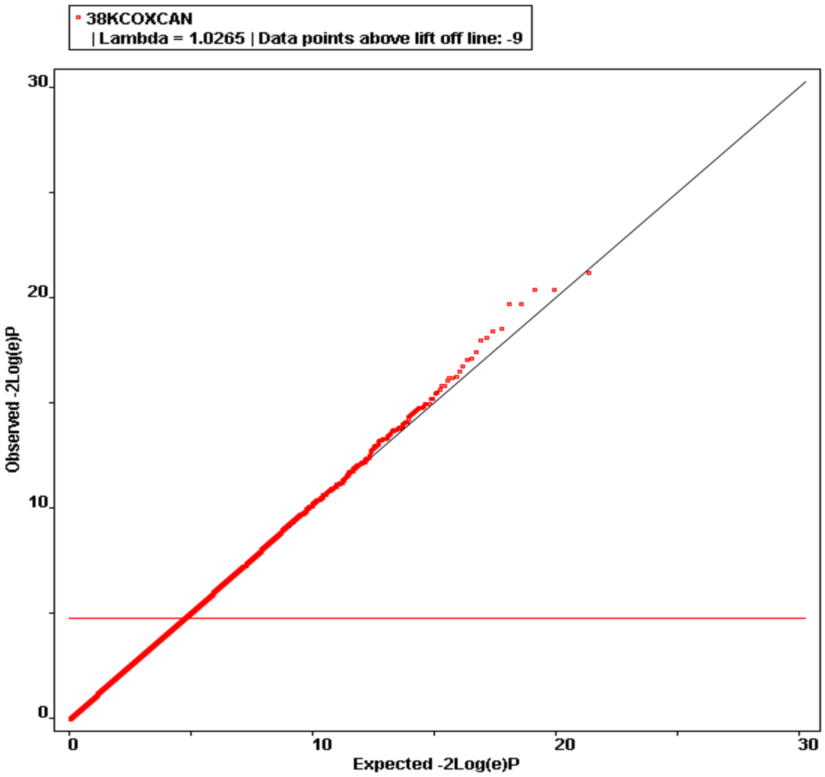
38KCOXCAN
| Lambda = 1.0265 | Data points above lift off line: -9
Observed -2Log(e)P
Expected -2Log(e)P
0
10
20
30

d Neurologic disease

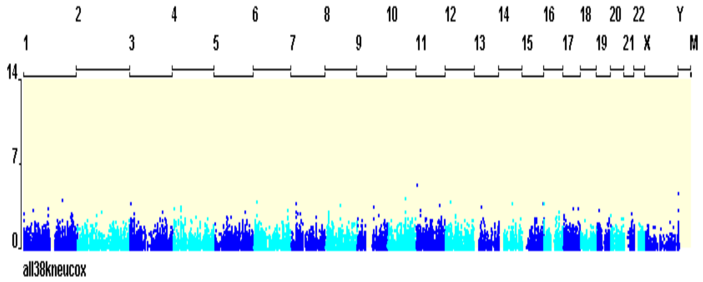

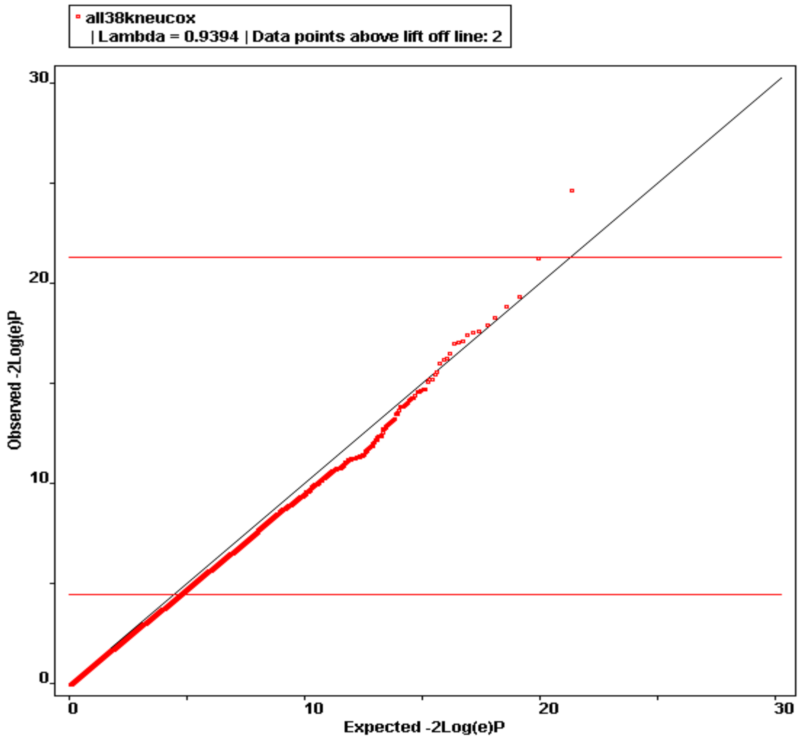

e Chronic kidney disease

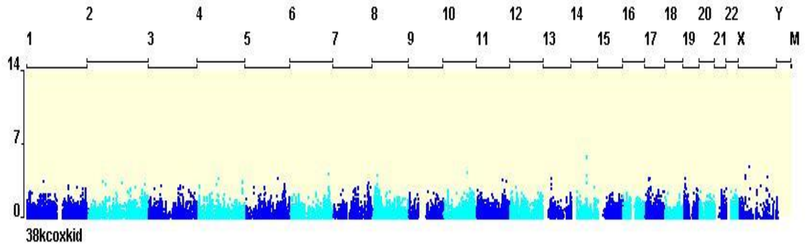

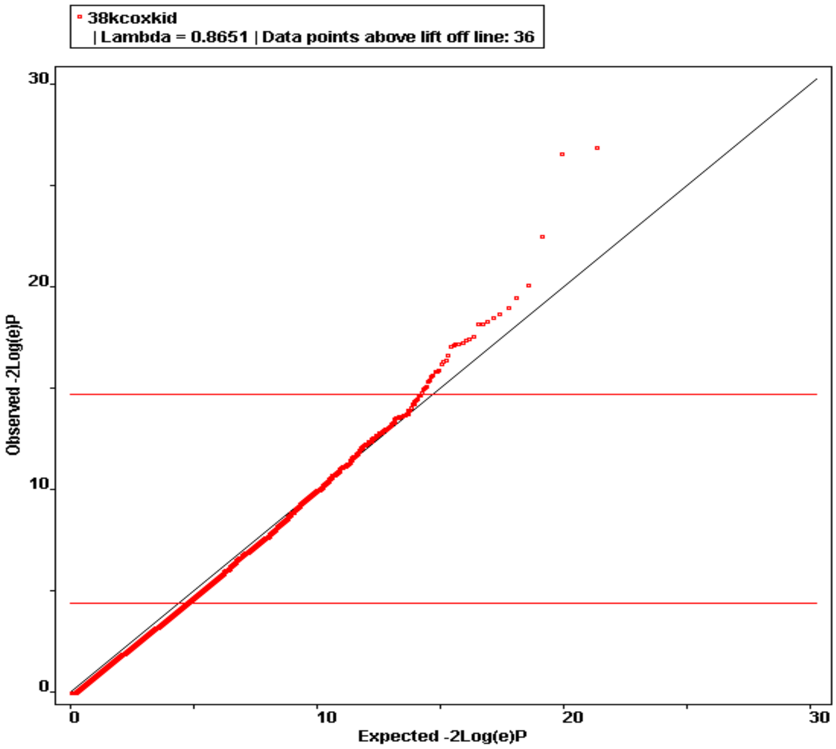

REFERENCES

37 Van Wieren-De Wijer, Diane BMA, et al. Interaction between the Gly460Trp α-adducin gene variant and diuretics on the risk of myocardial infarction. *Journal of hypertension*. **27.1**, 61-68 (2009).

38 Vliet-Ostaptchouk, Jana V., et al. Common variants in the type 2 diabetes KCNQ1 gene are associated with impairments in insulin secretion during hyperglycaemic glucose clamp. PLoS One. **7.3**, e32148 (2012).

39 Peters, Bas JM, et al. Genetic variability within the cholesterol lowering pathway and the effectiveness of statins in reducing the risk of MI. *Atherosclerosis*. **217.2**, 458-464 (2011).

40. Ikram, M. Arfan, et al. The Rotterdam Study: 2018 update on objectives, design and main results. *European Journal of Epidemiology*. **32.9**, 807-850 (2017).

41. Oeppen, Jim, and James W. Vaupel. Broken limits to life expectancy. 1029-1031 (2002).

42. Vermond, Rob A., et al. Incidence of atrial fibrillation and relationship with cardiovascular events, heart failure, and mortality: a community-based study from the Netherlands. *Journal of the American College of Cardiology*. **66.9**, 1000-1007 (2015).

43. Hillege, Hans L., et al. Prevention of Renal and Vascular End Stage Disease (PREVEND) Study Group. Urinary albumin excretion predicts cardiovascular and non-cardiovascular mortality in general population. *Circulation* **106.14**, 1777-1782 (2002).
